# Interstitial ectopic pregnancy: A rare case report

**DOI:** 10.1016/j.ijscr.2024.109529

**Published:** 2024-03-18

**Authors:** Majd Sabbagh, Nehad Othman, Salah Chaikha, Rehab Sukkar, Amal alToto, Lina alQudsi

**Affiliations:** a Department of Gynecology and Obstetrics, University Hospital of Obstetrics and Gynecology in Damascus, Faculty of Medicine, Damascus University, Damascus, Syria; b Faculty of Medicine, Damascus University, Damascus, Syria; c Faculty of Medicine, University of Algeria, Algeria; d Syrian Medical Association, Dar Al Shifa'a Hospital, Damascus, Syria

**Keywords:** Interstitial pregnancy, Diagnosis, Case report, Ectopic pregnancy, Tubal pregnancy

## Abstract

**Introduction:**

Interstitial pregnancies are a rare form of ectopic pregnancy with life threatening consequences. Thus, the aim of this report is to shed light on the importance of early diagnosis for optimal outcomes.

**Case presentation:**

Herein, we present a case of an interstitial ectopic pregnancy in a 31-year-old Syrian female who presented only with mild non-specific abdominal pain. The ultrasound showed a gestational sac in the right horn of the uterus non-communicating with the endometrial cavity suggesting an interstitial ectopic pregnancy. Cornual excision and salpingectomy were performed with laparotomy instead of laparoscopy due to resource-limited facilities. Follow-up with serum human chorionic gonadotropin β-HCG continued until the hormone levels became undetectable.

**Discussion:**

Interstitial ectopic pregnancies can present with non-classic symptoms. Ultrasonographic evaluation for lower abdominal pain in women at the first trimester is essential to detect interstitial ectopic pregnancies.

**Conclusion:**

Early diagnosis is key to prevent the life threatening progression of interstitial pregnancy, so this diagnosis should be kept in mind in women presenting with first trimester abdominal pain and/or vaginal bleeding.

## Highlights

• Interstitial ectopic pregnancy is the most life-threatening among tubal pregnancies.
• Interstitial pregnancies diagnosis is a challenge due to non-classic presentation.
• Patients can be asymptomatic or can present with non-specific abdominal pain.
• Ultrasonography plays a major role in the early diagnosis of interstitial pregnancy.
• Main treatments are medical with methotrexate or surgical.

## 1 Introduction

An ectopic pregnancy (EP) refers to the implantation of an embryo outside of the uterus [1]. The great majority of ectopic pregnancies implant in the fallopian tube (96 %). In addition, only 7.3 % of tubal pregnancies are interstitial making them the rarest among tubal pregnancies [2].

The most common symptom of EP is vaginal bleeding associated with pain and lower abdominal cramps. Interstitial pregnancy poses a serious diagnostic challenge due to late presentation, low sensitivity and low specificity of symptoms. Women with interstitial pregnancy could remain asymptomatic for several weeks because the interstitial portion of the tube can expand more than other tubal segments before rupture [3]. Interstitial ectopic pregnancies are life-threatening and associated with a 2–5 % mortality rate and a high risk of uterine rupture when compared to other tubal pregnancies [4].

The diagnosis is based upon transvaginal ultrasound and serum human chorionic gonadotropin (β-hCG). Interstitial pregnancy should be considered when we find eccentric implantation of the gestational sac at the fundal level of the uterus. [5]

The main risk factors associated with the incidence of ectopic pregnancy are prior ectopic pregnancy, prior tubal surgery, and prior pelvic/abdominal surgery. [6]

Management includes medical (with methotrexate) and surgical (cornuostomy or cornual resection by laparotomy or laparoscopy) treatments [3].

This case has been reported in line with the SCARE criteria [7].

We present a case report of a rare interstitial ectopic pregnancy in a 31-year old female who presented to the emergency department with non-specific lower abdominal pain in the first trimester. The early diagnosis prevented the progression to rupture of the ectopic pregnancy.

## 2 Case presentation

Our case report describes a 31-year-old female Syrian patient (gravida two, para one) of 8 ^+3^ weeks of gestation depending on the last menstrual period date, who walked into the emergency department presenting with mild lower abdominal pain and she was otherwise healthy.

Pain started four days before the presentation, it was dull, continuous and mild, it was localized in the lower abdomen with no radiations or exacerbating or relieving factors.

The patient did not report any underlying disease. She did not mention a history of smoking, alcohol, or drugs. Her surgical history only included a cesarean section four years ago, due to fetal distress, where she was not reported to have any uterine anomalies. She did not have a history of infertility and did not undergo assisted reproductive techniques. She did not report a history of gynecological surgeries or sexually transmitted diseases.

Vital signs of the patient were normal at the time of presentation. Physical examination was unremarkable. No abdominal rigidity, involuntary guarding, or severe tenderness was found. On bimanual pelvic examination, no adnexal or cervical motion tenderness was noticed.

**Fig. 1:**
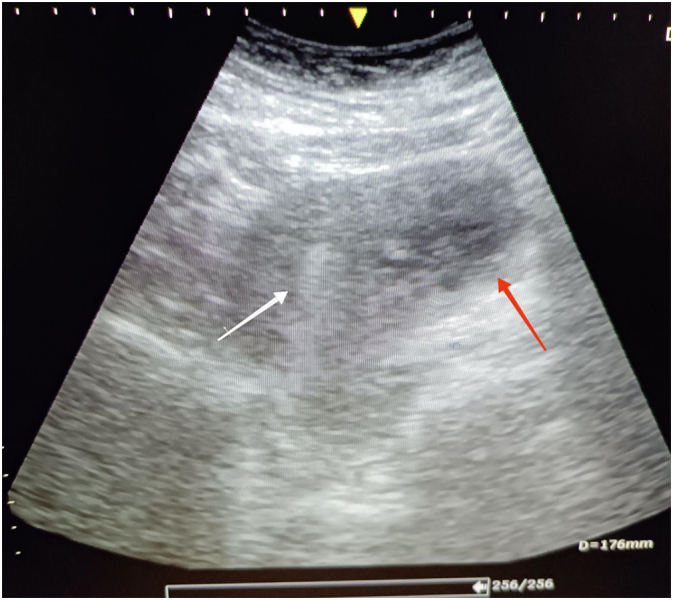
: Transabdominal ultrasound showing the ectopic pregnancy occupying the right uterine horn separated from the endometrium (White arrow: endometrium, red arrow: gestational sac).

**Fig. 2:**
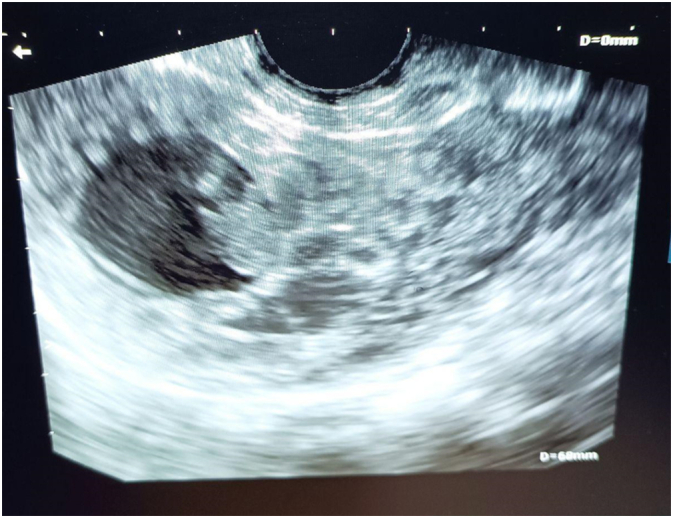
: Transvaginal ultrasound showing the gestational sac with an embryo inside.

Routine emergency department scan was conducted. Transabdominal and transvaginal ultrasound were performed and a gestational sac of 4.2 cm × 4 cm with an embryo with cardiac activity was observed occupying the right horn of the uterus and was not detected to be communicating with the endometrial cavity. No adnexal abnormalities were detected (Fig. 1, Fig. 2).

Laboratory tests showed no abnormalities but a positive pregnancy test.

Surgical intervention was decided and scheduled on the next day. Serum β-hCG level was 8735 mIU/ml.

**Fig. 3:**
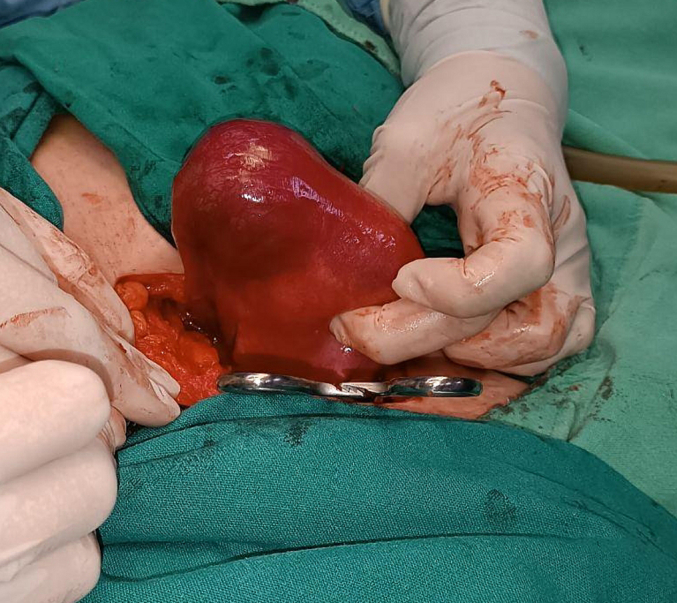
: The bulge of the interstitial ectopic pregnancy on the right side of the uterus.

**Fig. 4:**
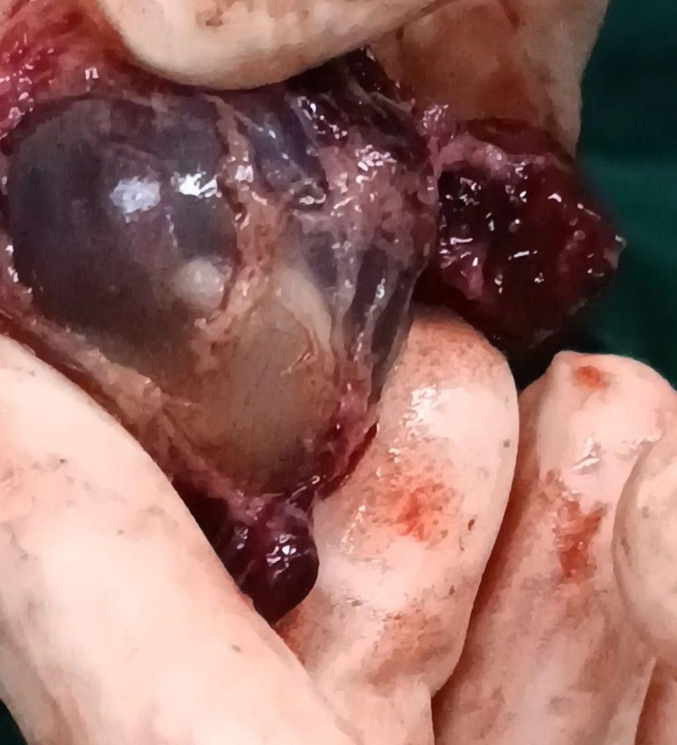
: Gestational sac with an embryo inside after surgery.

**Fig. 5:**
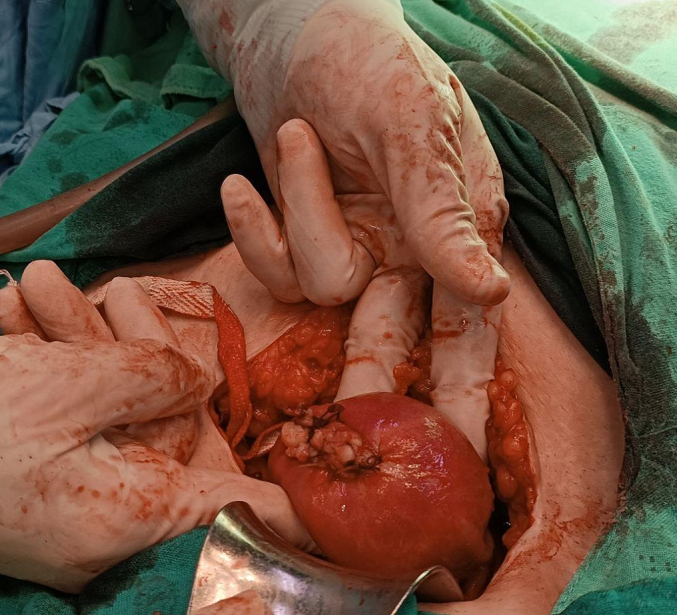
: The uterus after cornual resection.

Under general anesthesia, laparotomy was carried out and the diagnosis of an unruptured right interstitial pregnancy was confirmed (Fig. 3, Fig. 4). Right cornual resection with right salpingectomy were performed and hemostasis was achieved (Fig. 5). No complications were confronted in the postoperative period and the patient was discharged from the hospital after 24 h from surgery.

We performed β-HCG measurement after 48h from surgery and then repeated the test weekly until it became undetectable, which happened after 4 weeks from the surgery. Pathological findings were corresponding with the previously made diagnosis.

The patient was advised that close antenatal surveillance in subsequent pregnancies is necessary to monitor the risk of uterine rupture and decrease the incidence of recurrent cornual or interstitial ectopic pregnancies. It was also emphasized that cesarean delivery may be required in future pregnancies to reduce the risk of uterine rupture in labor.

## 3 Methods

This case has been reported in line with the SCARE criteria [7].

## 4 Discussion

We present a rare and diagnostically challenging case of an interstitial ectopic pregnancy.

Interstitial pregnancies are the rarest tubal pregnancies which were thought to make only 2–4 % of tubal pregnancies but now are thought to make 7.3 % of them [2]. The interstitial portion of the fallopian tube is the proximal segment that is embedded within the muscular wall of the uterus. The term intramural (interstitial) ectopic pregnancy refers to pregnancy developing outside the uterine cavity, with a gestational sac implanted into the interstitial part of the Fallopian tube, surrounded by a layer of the myometrium. The significance of interstitial pregnancy lies in the likelihood of delayed diagnosis due to its unique anatomic location [[8], [9], [10]]. Patients with interstitial/cornual ectopic pregnancy may have a sevenfold-higher mortality rate due to the fact that they rupture later and bleed more [11]. Although the maternal mortality rate associated with tubal pregnancy is decreasing, the mortality rate for interstitial pregnancies remains at 2.0 to 2.5 % because of misdiagnosis of these gestations as IUPs [12,13].

The major cause of ectopic pregnancy is disruption of normal tubal anatomy by factors such as infection, surgery, congenital anomalies, or tumors. Previous pelvic/abdominal surgery is a moderate risk factor for ectopic pregnancy [5]. Our patient only had a previous lower segment cesarean section. One unique risk factor for interstitial pregnancy is ipsilateral salpingectomy [14]. This particular risk factor was not present in our case.

Patients with ectopic pregnancies may present with vaginal bleeding and/or abdominal pain. It may be asymptomatic [15]. In our case the patient presented with nonspecific mild lower abdominal pain, which started four days before the presentation. The pain did not increase in severity and was dull, continuous, mild and localized in the lower abdomen.

On ultrasound examination, the key finding is that an interstitial pregnancy is extra-endometrial. As interstitial pregnancies advance, ultrasound may show a bulge at the interstitial region without a connection to the endometrial cavity; typically, there will be no myometrium surrounding a portion of the gestational sac. The interstitial section of the fallopian tube is evaluated in the transverse plane at the level of the uterine fundus and appears as a thin echogenic line lying between the lateral aspect of the endometrium and the uterine serosa. Some interstitial pregnancies may be misdiagnosed as intrauterine because they are partially implanted in the endometrium. A clue to correct diagnosis is its eccentric location and thin (<5 mm) myometrial mantle [16]. In our case, no communication with the endometrial cavity was detected.

The two main treatments of ectopic pregnancy are medical or surgical treatment. Medical treatment is preferred but surgery is required if any of the following exists: 1 - hemodynamically unstable patient, 2 - contraindications of methotrexate use, 3 - signs and symptoms of impending or ongoing rupture like abdominal and pelvic pain or hemoperitoneum, 4 - presence of fetal cardiac activity, serum β-hCG levels > 5000 mIU/mL, or EP > 4 cm in diameter [17].

In this patient due to the presence of fetal cardiac activity, the size of the gestational sac of >4 cm, β-hCG levels of 8735 mIU/mL and the presence of the abdominal pain suggestive of impending rupture, we chose surgical intervention not medical treatment.

The preferred surgical intervention is laparoscopic cornuostomy as it causes less tubal damage and better future pregnancy outcomes but it is advised to perform cornual resection in cases of interstitial pregnancies of advanced gestational age and/or when ectopic size was >4 cm in diameter. Cornual resection is generally an effective and quite safe procedure, but it can cause significant bleeding. Cornual resection by laparotomy or laparoscopy could cause uterine rupture in subsequent pregnancies [18,19]. In our settings, laparotomy was conducted instead of laparoscopy due to resource-limited facilities and cornual resection was performed.

Patients are advised that future pregnancies after cornual resection should be monitored with ultrasound at 5 to 6 weeks of gestation to exclude recurrent ectopic pregnancy and are advised to be delivered by elective cesarean section to decrease the risk of uterine rupture at labor [3,18].

## 5 Conclusion

The lack of suspicion and the late presentation of patients with non-classic symptoms can lead to the delayed diagnosis and severe and potentially fatal outcomes of interstitial pregnancies.

Therefore, the key to prevent that is to keep this diagnosis in mind during the evaluation of patients with non-classic presentations, like first trimester vague abdominal pain.

## Ethical approval

No ethical approval needed for case report in our Institution.

## Funding

The authors received no funding regarding the publication of this article.

## Author contribution

Majd Sabbagh corresponding author and Guarantor contributed in writing the paper and data analysis and interpretation.

Nehad Othman contributed in writing the paper and data analysis and interpretation.

Prof. Salah Chaikha contributed in data analysis and interpretation.

Rehab Sukkar contributed in data collection.

Amal alToto contributed in data collection.

Lina alQudsi contributed in study concept.

## Guarantor

Majd Sabbagh.

## Research registration number

This case report is not first in man.

## Consent

Written informed consent was obtained from the patient to publish this report.

## Conflict of interest statement

The authors declare that there is no conflict of interest to be reported.
